# Seroprevalence and associated risk factors for bovine leptospirosis in Egypt

**DOI:** 10.1038/s41598-024-54882-4

**Published:** 2024-02-26

**Authors:** Abdelfattah Selim, Mohamed Marzok, Hattan S. Gattan, Abdelhamed Abdelhady, Mohamed Salem, Abdelrahman M. Hereba

**Affiliations:** 1 Department of Animal Medicine (Infectious Diseases), Faculty of Veterinary Medicine, Benha University, Toukh, 13736 Egypt; 2 Department of Clinical Sciences, College of Veterinary Medicine, King Faisal University, 31982 Al-Ahsa, Saudi Arabia; 3 Department of Surgery, Faculty of Veterinary Medicine, Kafr El Sheikh University, Kafr El Sheikh, Egypt; 4 Department of Medical Laboratory Sciences, Faculty of Applied Medical Sciences, King Abdulaziz University, Jeddah, Saudi Arabia; 5 Special Infectious Agents Unit, King Fahad Medical Research Center, King AbdulAziz University, Jeddah, Saudi Arabia; 6 Department of Parasitology and Animal Diseases, National Research Center, Giza, Egypt; 7 Department of Biomedical Physics, Medical Research Institute, Alexandria University, Alexandria, Egypt; 8 Department of Medicine and Infectious Diseases, Faculty of Veterinary Medicine, Cairo University, 12613 Cairo, Egypt; 9 Department of Microbiology, College of Veterinary Medicine, King Faisal University, 31982 Al-Ahsa, Saudi Arabia

**Keywords:** *Leptospira* spp, Serology, Risk factors, Cattle, Egypt, Bacteria, Risk factors

## Abstract

Leptospirosis is caused by pathogenic bacteria of the genus *Leptospira* and is one of causative agents of reproductive problems leading to negative economic impact on bovine worldwide. The goal of this study was to investigate the seroprevalence of *Leptospira* spp. in cattle in some governorates of Egypt's Nile Delta and assess the risk factors for infection. A total of 410 serum samples were collected from cattle and examined using microscopic agglutination test. The overall seroprevalence was 10.2% and the most prevalent serovars were Icterohaemorrhagiae, Pomona and Canicola. In addition, the potential risk factors were associated *Leptospira* spp. infection were age, herd size, history of abortion, presence of dogs and rodent control. Thus, leptospirosis is common in dairy cattle in the Nile Delta and  the presence of rodents in feed and dog-accessible pastures increases the risk of *Leptospira* spp. infection among animals.

## Introduction

Leptospirosis is a global zoonotic threat that poses a global public health problem due to its high mortality and morbidity rates^1,2^. The disease is caused by pathogenic bacterium of genus of *Leptospira*, which occurs primarily in tropical and subtropical countries where humid climates and high temperatures favor bacterial growth^3,4^.

This pathogen spreads mostly by direct or indirect exposure to urine of the principal reservoirs (rodents) and other animals. Moreover, the bacterium persist in renal tissue of infected animals for variable periods and shedding in urine causing contamination to environment^5,6^.

In cattle, infection can occur directly through contaminated urine, post-abortion secretions, infected placenta, or sexual contact. However, indirect transmission plays a significant role in infection dissemination^7,8^. Bovine leptospirosis is characterized mostly by reproductive losses such as abortions and stillbirths, as well as poor weight growth, mastitis, and reduction in milk yield. Nevertheless, laboratory testing, primarily serological techniques, are used to support the diagnosis^9,10^.

Human contract *Leptospira* by coming into contact with infected urine or by visiting a urine-contaminated environment^11^. Mucosal and conjunctival tissues as well as scratches and cuts are common entry points^12^. Human infections can cause severe, potentially fatal illnesses, but in most cases remain asymptomatic or cause mild ailments. This disease causes non-specific signs and symptoms, including fever, headaches, dry coughs, abdominal discomfort, myalgia, and nausea^13^.

The epidemiology of leptospirosis and the incidence of the disease in the cattle herds have both been found to be significantly influenced by the presence of dogs on rural farms^14^. Cattle positive serology has shown that rodents that have direct contact with cattle feeding are another significant risk factor^15^.

For a definitive diagnosis of leptospirosis, laboratory testing is required. Dark-field microscopy can be used to show the organism in the blood, urine, or cerebrospinal fluid^16,17^. The ELISA is used as a first screening test and is a crucial piece of clinical immunology equipment. For the diagnosis of leptospirosis, additional tests are employed, such as the microscopic agglutination test, fluorescent antibody test, indirect hemagglutination test, radial immunoassay, complement fixation test, and PCR^18–20^. The most often used laboratory technique for *Leptospira* diagnosis is ELISA, which is also commercially accessible. PCR is less frequently employed. ELISA can identify antibodies from the second weeks of infection forward and has higher sensitivity and specificity than the microscopic agglutination test^21^.

The global prevalence of animal leptospirosis with wide ranges from 2 to 46% according to animal species^22,23^, this variation might be climatic changes and diagnostic techniques.

In Egypt, the previous researches focused on leptospirosis in people exposed to animals. The ELISA test used to identify Leptospiral antibodies in people with unexplained acute febrile sickness and hepatitis^24^. However, little information is known on the prevalence of leptospirosis in cattle across Egypt's key cattle-producing provinces, notably the Nile Delta province, which includes Dakahlia Governorate^25^.

This study aimed to identify seroprevalence of *Leptospira* spp. infection and to assess risk factors associated with *Leptospira* infection in dairy cattle in northern Egypt.

## Materials and methods

### Ethical statement

Benha University's ethics committee for animal research approved the study's methodology and techniques. All cattle owners provided informed consent to participate in the study. The Faculty of Veterinary Medicine's ethics committee guaranteed that all operations followed all applicable rules. The ARRIVE criteria were followed throughout the study process.

### Study site

**Figure 1:**
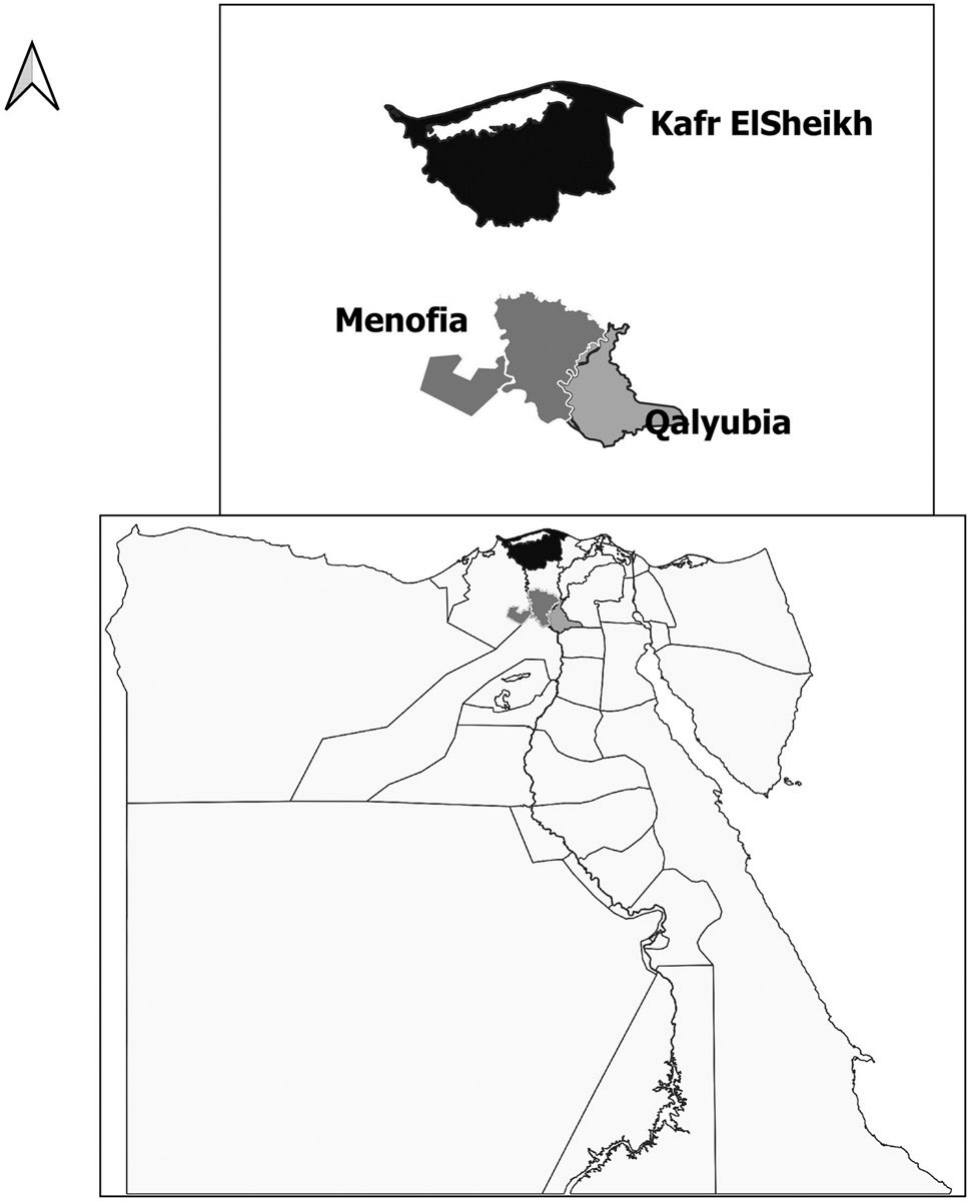
MAP illustrated the governorates under the study (MAP generated by QGIS software).

This study was performed during the period of March 2021 to February 2022 and cover three governorates (Kafr ElSheikh, Menofia and Qalyubia) situated at Nile Delta of Egypt, Fig. 1. The selected governorates are located at latitudes 31° 06′ 42″ N, 30.52° N, and 30.867° N, respectively, and at longitudes 30° 56′ 45″ E, 30.99° E, and 31.028° E.

A hot desert climate dominates the Nile Delta in general, but in its northernmost part, which is also the wettest region in Egypt, it has relatively moderate temperatures with a high of 31 °C in the summer, as is the case with all of the northern coast of Egypt.

### Sample design and sampling

The sample size were determined using the following formula according^26^ using the procedure for simple random sampling:

$${\text{N}} = Z^{{{2}*}} P\left( {{1} - P} \right)/d^{{2}}$$

where n is the sample size, *P* is the predicted prevalence 50%, *Z* = 1.96 with 95% confidence level, and *d* is the absolute error 5%. The calculated number of samples was 384 and increased to 410 to increase the precision.

In order to obtain serum, cattle blood samples were collected using vacuum tubes without anticoagulant through punctured the jugular vein and centrifuged at 3000 xg for ten minutes. the serum was stored at − 20 °C in 1.5-mL Eppendorf tubes till serological examination was completed.

### Data collection

Cattle owners provided the database with their individual information to identify potential risk factors for leptospirosis seropositivity. At the time of blood sampling, each participant filled out a questionnaire. A number of variables were selected: (1) location (Kafr ElSheikh, Menofia and Qalyubia), (2) age (2, 2–3, and > 3 years), (3) sex (male and female), (4) herd size (50, 50–75, and > 75), (5) gestation status (pregnant and non-pregnant), (6) history of abortion (yes or no), (7) presence of dogs (yes or no), and 8) rodent control (yes or no). The samples were collected randomly from individual farmer, two medium herds and one large herd.

### Serological diagnosis

In accordance with the recommendations of the World Organization for Animal Health (OIE), the serological diagnosis was carried out using a microscope equipped with a dark field condenser to conduct the microscopic agglutination test (MAT) as described by^27^. The panel of antigens utilized in this investigation contained seven common strains, taking into account the most common serovars of *Leptospira interrogans* in the country: Canicola, Hardjo, Pomona, Icterohaemorrhagiae, Grippotyphosa, Bratislava, and Copenhageni. A dilution of 1:50 was used for the initial testing of sera samples, and those with an agglutination level equal to or greater than 50% were further diluted. The final titration was calculated as the dilution at which 50% agglutination was detected. A titration of 1:50 indicated that the animals had been exposed to the causative agent. Titrations of 1:100 were regarded as positive for *Leptospira* infection.

### Statistical analysis

The data from the questionnaires were analysed to identify potential risk factors for leptospirosis seropositivity. The analysis was done in two stages: univariate and multivariate. In the univariate analysis, each independent variable was crossed with the dependent variable (seropositivity), and those with a chi-square test *P*-value < 0.20 were subjected to multivariate logistic regression analysis^28–33^. A correlation analysis was used to confirm collinearity between independent variables; for those variables with substantial collinearity (correlation coefficient > 0.9). The statistical analysis was performed by SPSS software ver. 24 (IBM < USA).

## Results

In total, out of 410 animals examined, 42 tested seropositive, indicating a seroprevalence of 10.2% (95% CI 7.66–13.55). The analysis of the identified sera revealed that serovar Icterohaemorrhagiae was the most prevalent at 2.9% (95% CI 1.68–5.05), while Copenhageni exhibited the lowest occurrence with 0.24% (95% CI 0.04–1.36), Table 1.

**Table 1:** The prevalence of *Leptospira* in relation to different serovars.

Serovar	Total No of examined sample	No of positive (%)	95%CI
Icterohaemorrhagiae	410	12 (2.9)	1.68–5.05
Canicola	410	8 (1.9)	0.99–3.8
Hardjo	410	6 (1.5)	0.67–3.15
Pomona	410	9 (2.2)	1.16–4.12
Bratislava	410	4 (0.98)	0.38–2.49
Grippotyphosa	410	2 (0.49)	0.13–1.76
Copenhageni	410	1 (0.24)	0.04–1.36
Total	410	42 (10.2)	7.66–13.55

The univariate analysis for the variables associated to seropositivity for any *Leptospira* spp. serovar in cattle were presented in Table 2. The seroprevalence revealed non-significant (*P* > 0.05) association between locality, sex and gestation status and *Leptospira* seropositivity.

**Table 2:** Prevalence of *Leptospira* spp. in cattle in relation to different variables.

Variables	categories	animals sampled	No of positive animals	Prevalence%	95%CI	Statistic
Locality	Kafr ElSheikh	150	21	14.0	9.34–20.46	χ2 = 5.043 df = 1 *P* = 0.080
	Menofia	125	13	10.4	6.18–16.98	
	Qalyubia	135	8	5.9	3.04–11.26	
Age	< 2	70	2	2.9	0.79–9.84	χ2 = 8.348 df = 2 *P* = 0.015*
	2–5	220	21	9.5	6.33–14.16	
	> 5	120	19	15.8	10.37–23.41	
Sex	male	51	8	15.7	8.17–28.01	χ2 = 1.876 df = 1 *P* = 0.171
	female	359	34	9.5	6.86–12.94	
Herd size	< 50	290	7	2.4	1.17–4.89	χ2 = 69.489 df = 2 *P* < 0.0001*
	50–75	85	22	25.9	17.76–36.09	
	> 75	35	13	37.1	23.16–53.66	
Gestation status	Pregnant	150	14	9.3	6.24–14.85	χ2 = 0.006 df = 1 *P* = 0.940
	non-pregnant	209	20	9.6	7.28–15.38	
History of abortion	Yes	116	19	16.4	10.17–20.81	χ2 = 9.540 df = 1 *P* = 0.002*
	No	243	15	6.2	4.47–11.05	
Presence of dogs	Yes	155	29	18.7	13.35–25.58	χ2 = 19.426 df = 1 *P* < 0.0001
	No	255	13	5.1	3–8.53	
Rodent control	Yes	192	11	5.7	3.23–9.97	χ2 = 8.005 df = 1 *P* = 0.005*
	No	218	31	14.2	10.2–19.48	
Total		410	42	10.2	7.66–13.55	

*The result considered significant if *P* < 0.05.

χ2: Chi-square, df: degree of freedom , *P*: P value.

The seroprevalence rose with age and was substantially (*P* < 0.05) higher in cattle over 5 years old (15.8%), particularly in those raised in large herd sizes (37.1%). Furthermore, *Leptospira* seroprevalence in cattle increased significantly (*P* < 0.05) in animals with a history of miscarriage (16.4%), in animals living with dogs (18.7%), and in homes without rodent management (14.2%), Table 2.

The variables with *P* < 0.2 in univariate analysis were included in multivariate logistic regression model. The variables were identified as risk factors in multivariate model for *Leptospira* seropositivity were age more than five years (OR 7.24, *P* = 0.027), large herd size more than 75 (OR 30.53, *P* < 0.0001), animal with history of abortion (OR 1.49, *P* = 0.036), presence of dogs (OR 6.32, *P* < 0.0001) and absence of rodents control (OR 2.03, *P* = 0.010), Table 3.

**Table 3:** Multivariate analysis for risk factors associated with *Leptospira* spp. infection.

Variable	B	S.E	OR	95% CI for OR	*P* value
Age					
2–5	1.638	0.876	5.14	5.14–28.63	0.061
> 5	1.980	0.893	7.24	1.26–41.73	0.027
Herd size					
50–75	2.724	0.498	15.24	5.75–40.42	< 0.0001
> 75	3.419	0.628	30.53	8.91–10.62	< 0.0001
History of abortion					
Yes	0.398	0.438	1.49	0.63–3.51	0.036
Presence of dogs					
Yes	1.844	0.433	6.32	2.71–14.77	< 0.0001
Rodents control					
No	0.706	0.439	2.03	0.86–4.79	0.010

*B* Logistic regression coefficient, *SE* Standard error, *OR* Odds ratio, *CI* Confidence interval.

## Discussion

Leptospirosis is a global zoonotic threat and information on the disease's epidemiology and the variables that contribute to its incidence is very important to improve the control level of leptospirosis^34^. In particular, few studies to our knowledge have been considered the epidemiological situation of leptospirosis in cattle in Dakhalia governorates but no data about its prevalence in other governorates of Nile Delta. Therefore, one of the major aim of this study is determination the seroprevalence of *Leptospira* spp. in cattle in three Egyptian governorates and assess its associated potential risk variables.

In this study, the seroprevalence of *Leptospira* spp. in cattle raising the three studied governorates in Nile Delta (Kafr ElSheikh, Menofia and Qalyubia) was 10.2% (95% CI 7.66–13.55). In another Nile Delta governorate, cattle seroprevalence was estimated to be 39.33%^25^. As a result, the findings emphasise the significance of this disease in the country and the necessity to develop effective control measures to lower its incidence.

However, the *Leptospira* spp. seroprevalence is higher in some countries such as 81.7% in Northeastern Malaysia^35^, 89.9% in Poland^36^, 88.2% in Mexico^37^, 81% in Chile^38^, and 87% in India^39^.

Alternatively, lower prevalences have been reported in some countries, it was 3% in North Eastern India^40^, 3.2% in Poland^41^, 13% in Tanzania^42^, 20.3% in Sri Lanka^43^, 31.3% in Brazil^44^, and 24.48% in southwestern Ethiopia^45^.

Several factors may contribute to this variation, including geography, husbandry practices, management, sampling and diagnostic method, natural immunity, and disease resistance^9,14,30,32,33,45–47^. In addition, high densities of infected cows with *Leptospira* spp. might lead to environmental contamination and disease spreading since they could serve as reservoirs and spread infection to other animals residing in the same habitat^48^.

Interestingly, the most prevalent serovars among examined cattle in the present study were Icterohaemorrhagiae (2.9%), Pomona (2.2%) and Canicola (1.9%). These findings are in accordance with previous findings reported by^49^ and^50^, they found the most common serovars in cattle Pomona and Icterohaemorrhagiae. Moreover, Icterohaemorrhagiae and Pomona serogroups are associated to animal interaction with various animal species that serve as reservoirs for the diseases^51^.

In the present study, the seroprevalence of *Leptospira* spp. did not varied between studied governorates because all of them situated in the Nile Delta and have the same climatic features and topographic characters^52^. Moreover, Marzok, et al.^52^ found that the most prevalent serovars in Egypt was Icterohaemorrhagiae, Canicola and Pomona.

Similar to previous findings of dos Santos, et al.^44^, but in contrast with findings of Parvez, et al.^53^, the seroprevalence of *Leptospira* spp. increased significantly with age. In addition, in an Indian investigation, Sudharma and Veena^54^ observed that the seroprevalence was not correlated with animal age. This might be attributable to the fact that exposure to *Leptospira* becomes more common as old cattle, and that seropositivity can remain for a very long period^1,25^.

The present findings revealed that the females were more seropositivity for *Leptospira* spp. than males, this consistent with previous findings of El-Deeb, et al.^25^ and Ijaz, et al.^55^. However, many previous studies have shown that males are more likely to contract leptospirosis than females without a significant variation^56,57^. There is no clear explanation for these findings and reported differences in relation to sex^57^. The result of present study might be contributed to most of the samples examined were collected from female cows which give its potential influence.

*Leptospira* spp. seroprevalence significantly increased in large herd size in accordance with prior findings of Benseghir, et al.^58^. This finding may be explained by inadequate sanitation facilities, difficulty in monitoring hygienic practices on large herds compared to small herds and Leptospiral infection spread rapidly in overcrowded farms which have poor management and sanitation application^4,35,44,55^.

In the current study, the prevalence of *Leptospira* spp. was higher in cattle suffered from history of abortion or second semester of pregnancy. The findings confirm previous reports that *Leptospira* spp. present chronically in bovines and can lead to sexual dysfunction, low fertility, and abortion^59,60^.

The presence of dogs increased the prevalence of *Leptospira* spp. in cattle, which come in agreement with previous findings of Fávero, et al.^49^. Moreover, *Leptospira* spp. were more prevalent in cattle raising farm which have poor management and rodent control. Similar findings were concluded by Motto, et al.^42^. Rodents are mostly recognized epidemiologically for spreading various pathogenic *Leptospira* and contaminating pasture^61^, and as a result, animals may contract leptospirosis during grazing^62^.

## Conclusion

The results of present study confirmed that *Leptospira* spp. present among cattle in Nile Delta of Egypt, contributed as cause of abortion in pregnant animals. The multivariate logistic regression model identified age, herd size, history of abortion and control of rodents as potential risk factors for *Leptospira* spp. infection. The identification of species and biovars, the understanding of transmission cycles, and the implementation of preventative and control measures are critical, particularly for dairy cows, as well as identifying alternatives to management practices that could spread disease to people or animals.

## Data Availability

All data generated or analysed during this study are included in this published article.
